# Dissimilarity in the Frequency of Venous Thromboembolism Risk Factors among Studies, a Commentary

**Published:** 2018-01-18

**Authors:** Meghdad Sedaghat, Mahsa Soltani, Mehrdad Solooki

**Affiliations:** 1 Department of Internal Medicine, Imam Hossein Hospital, Shahid Beheshti University of Medical Sciences, Tehran, Iran.; 2 Department of Pulmonary and Critical Care Medicine, Imam Hossein Hospital, Shahid Beheshti University of Medical Sciences, Tehran, Iran.

**Dear Editor;**

Venous Thromboembolism (VTE) is the 3^rd^ most prevalent vascular disease behind myocardial infarction and cerebrovascular ischemic attack (1). This disorder has received attention from health policy makers because of its major complications including recurrent VTE, post thrombotic syndrome, sudden cardiac death and high mortality rate (2). In the United States, VTE was reported in approximately 201000 cases annually, 25% of which expired within 7 days after diagnosis and 22% of mortalities did not have a definitive diagnosis. Despite the progression in diagnosis and treatment of VTE since 1979, its incidence did not decrease dramatically. This gap declares that VTE risk factors, especially transient ones, have not been detected completely (3).

Obesity, history of VTE, family history of VTE, recent surgery, malignancy, myeloproliferative disorders, trauma, pregnancy, post-menopausal hormone therapy, hereditary syndromes like anti phospholipid syndrome (APS), chronic kidney disease (CKD), chronic obstructive pulmonary disease (COPD), blood transfusion and older age are determined as major risk factors. These factors can be categorized into two major subgroups as intrinsic and predisposing. Recent investigations focused on predisposing ones, which can be justified (2, 4).

Designing a cross sectional study in Imam Hossein Hospital, Tehran, Iran, from 2016 to 2017, we found that inactivity due to disability (30.9%), smoking (29.3%), and active malignancy (18.1%), were the most prevalent transient risks factor of VTE in our sample, respectively. Similar to our results, Kesieme et al. (5) and Cushman et al. (2) declared that VTE is more diagnosed in the elderly. Park MS et al. (3) introduced recent surgery, trauma (73%), and disability to walk (62%) as major independent risk factors of VTE. Fuji T et al. (6) introduced malignancy, recent infectious disease, and obesity as the factors predisposing patients who were admitted for orthopedic surgery to VTE.

As can be seen, despite the risk factors of VTE being the same in various studies, their frequency varied between the studies. Population and cultural characteristics and various habits may have an effect in this regard. Therefore, it is suggested to perform a multi-center, comprehensive study considering all the racial and ethnic in order to have a correct pattern of the frequency of predisposing factors of this disease in the Iranian population for health and prevention programs.
